# Evaluating the Biological Effects of Bisphenol A Leaching During Clear Aligner Therapy: An Umbrella Review of Systematic Reviews and Meta-Analyses

**DOI:** 10.7759/cureus.88704

**Published:** 2025-07-24

**Authors:** Noyonika Mallik, Biswaroop Mohanty, Sanghamitra Jena, Bhagabati Dash, Nivedita Sahoo

**Affiliations:** 1 Department of Orthodontics and Dentofacial Orthopaedics, Kalinga Institute of Dental Sciences, KIIT (Deemed to be University), Bhubaneswar, IND

**Keywords:** bisphenol a, bisphenol a-glycidyl methacrylate, clear aligner appliances, clear aligner therapy (cat), effects on general health

## Abstract

The rising demand for aesthetic and comfortable orthodontic treatments has led to the widespread use of clear aligner therapy (CAT), a modern alternative to traditional braces. This umbrella review examines the biological effects of bisphenol A (BPA), a synthetic compound commonly found in clear aligner materials, focusing on its potential health risks. BPA is known for its estrogen-mimicking properties and has been associated with endocrine disruption and increased cancer risk. This review synthesizes findings from systematic reviews and meta-analyses to evaluate BPA leaching in aligner therapy and its implications for patient safety. A total of 25 studies were screened, with three high-quality systematic reviews included in the final analysis. In vitro studies generally reported minimal cytotoxicity and weak estrogenic effects, while clinical trials suggested higher BPA levels due to the dynamic oral environment. Risk of bias was assessed, and forest and funnel plots indicated consistent findings with low immediate toxicity but raised concerns regarding long-term hormonal effects. Given these findings, further research is recommended to assess BPA exposure under real oral conditions and to develop BPA-free aligner materials. This review underscores the importance of biocompatibility in orthodontic materials and the need for rigorous safety assessments to protect patient health.

## Introduction and background

Evolution of orthodontic appliances

Orthodontics dates back to ancient Egypt, where crude metal bands and catgut were used. Practical orthodontic appliances emerged in the late 18th century. Traditional labial (buccal) appliances remain prevalent, while lingual appliances gained popularity due to their aesthetic appeal, though adaptation challenges limited their use [1,2]. The introduction of Invisalign™ in 1997 offered a more aesthetic and removable alternative [3]. Adult orthodontic treatments have grown in popularity, with many opting for lingual or Invisalign™ methods. Studies indicate that women under 40 years widely prefer lingual over buccal for professional and aesthetic reasons [4,5]. Additionally, many females aged 20-30 years favor Invisalign™ due to its aesthetic and functional advantages [6,7].

Clear aligner therapy

The demand for aesthetic and comfortable orthodontic solutions has led to the development of clear aligner therapy (CAT) [8]. Initially conceptualized in 1946 by Kesling [9], who suggested a series of thermoplastic positioners for gradual tooth movement, the concept was refined in 1997 by Align Technology© (Santa Clara, CA), making it a viable treatment option. Originally used for minor irregularities, research advancements have expanded CAT's application to complex cases, including extractions [10]. The evolution of materials, manufacturing, and computerized tooth movement planning has significantly improved aligner technology, allowing for the treatment of mild to severe malocclusions [11]. CAT involves a sequence of clear plastic trays that fit snugly over the teeth and are replaced every one to two weeks to achieve planned movements. Despite variations among commercial systems, all aligners use thermoformed plastic materials [12].

Aligner Materials

Aligners are primarily composed of polymers, including polyethylene (PE), polyethylene terephthalate (PET), polyethylene terephthalate glycol (PETG), polyurethane (PU), and polypropylene (PP). Some common aligner materials are presented in Table 1 [13]. Bisphenols are frequently used to enhance plastic hardness and clarity [14]. Among aligner materials, Smart Track polyurethane has exhibited the highest bisphenol A (BPA) and bisphenol S (BPS) release [15].

**Table 1:** Materials used in various clear aligners

Aligner system	Material used	Manufacturer (company name)	Headquarters
Duran®	Polyethylene terephthalate glycol (PETG)	Scheu Dental GmbH	Iserlohn, Germany
Biolon®	Polyethylene terephthalate (PET)	Dreve Dentamid GmbH	Unna, Germany
Zendura®	Thermoplastic polyurethane (TPU)	Bay Materials LLC (Brand: ZenduraDental)	Fremont, CA, USA
Invisalign®	SmartTrack® (multilayer polyurethane + copolyester blend)	Align Technology Inc.	San Jose, CA, USA
ClearCorrect®	Zendura® polyurethane	ClearCorrect (a subsidiary of Straumann Group)	Round Rock, TX, USA
MTM® Clear Aligner	Raintree Essix ACE® (polyurethane)	Dentsply Sirona	York, PA, USA
Fantasmino®	Polyvinyl chloride (PVC)	Ortolan S.r.l.	Pompei, Napoli, Italy
Nuvola®	Polyethylene terephthalate glycol (PETG)	GEO Orthodontic	Rome, Italy

Bisphenol A (BPA) and Its Effects

BPA, a synthetic compound, is known for its estrogen-mimicking properties [16]. It disrupts hormonal balance by binding to globulins that regulate sex hormones, potentially affecting reproductive health, metabolism, and immune function. BPA exposure has been linked to endocrine disruptions, sperm production issues, and developmental abnormalities like midfacial hypoplasia, molar-incisor hypomineralization (MIH), hypodontia, or microdontia. It also influences the thyroid hormone axis, contributing to conditions like hypothyroidism [17]. Studies associate BPA exposure with various cancers, including breast, prostate, and lung cancers. Regulatory bodies have set safety limits for BPA exposure, with the European Food Safety Authority reducing the tolerable daily intake to 0.2 nanograms/kg bw/day in 2023 [18].

Health Risks of BPA

BPA affects biological processes through intracellular signaling and gene regulation. It activates pathways that influence cell proliferation, enzyme function, and oxidative stress responses. BPA can bind to estrogen receptors (ERα and ERβ), influencing estrogenic activity. Additionally, it interacts with androgen receptors, affecting reproductive functions. Studies suggest BPA's role in cancer development by modulating oncogenic pathways. While several studies have investigated BPA exposure through food and general consumer products, there is a lack of focused reviews examining BPA release specifically from orthodontic materials such as clear aligners. Understanding BPA’s biological effects in this context is crucial for evaluating its risks, guiding material selection, and ensuring patient safety without compromising the benefits of modern aligner technologies.

## Review

Methodology

This study was conducted as an umbrella review, which synthesizes evidence from multiple systematic reviews to provide a comprehensive overview of the existing literature. Umbrella reviews are particularly valuable for decision-makers who require high-level, consolidated evidence [19]. The protocol for this review was registered on PROSPERO before the commencement of the study, under the registration ID CRD42024506580.

The primary objective of this study was to evaluate the biological effects of BPA released from clear aligners in orthodontic patients. The research question was structured using the PICO (Population, Intervention, Comparison, and Outcome) framework. The population included patients undergoing orthodontic treatment with clear aligners, including individuals presenting with various types of malocclusions. The intervention involved the use of clear aligners, with BPA levels measured in blood samples to assess systemic exposure. The comparator group consisted of patients receiving orthodontic treatment without the use of clear aligners.

The outcomes of interest were the long-term BPA levels detected in patients and their potential impact on overall health. Studies were included if they were systematic reviews or meta-analyses published from 2019 onward that examined BPA release from clear aligners. Specifically, the included systematic reviews exhibited substantial heterogeneity in the following areas: study design (a mix of in vitro, in vivo, and limited human studies); measurement techniques (BPA release was assessed using gas chromatography-mass spectrometry (GC-MS), high-performance liquid chromatography (HPLC), or ELISA); material types (e.g., PETG, polyurethane, PVC); exposure conditions (differences in pH, temperature, duration of aligner immersion, and simulated oral environments); and outcome measures. Additionally, few studies directly linked BPA exposure to biological or systemic effects, making pooled synthesis difficult.

Eligible studies also included investigations into the materials used in clear aligner therapy or patient health outcomes related to BPA exposure. Only articles published in English were considered for inclusion. Studies were excluded if they were non-review articles, such as original research articles, case reports, editorials, or letters to the editor. Reviews published in languages other than English were also excluded. A comprehensive literature search was conducted across three major databases: PubMed, Scopus, and Web of Science. The search strategy incorporated both general keywords and Medical Subject Headings (MeSH) terms relevant to BPA and clear aligners. Boolean operators "AND" and "OR" were applied to refine the search results and maximize the chance of retrieval of relevant articles. To ensure comprehensive coverage, conference proceedings and gray literature were also reviewed for potentially eligible studies.

Two reviewers independently screened the titles and abstracts using Rayyan QCRI, a web-based platform designed to facilitate blinded review and conflict resolution in systematic reviews. Full-text screening was then conducted for eligible articles based on predefined inclusion and exclusion criteria. Discrepancies were resolved through discussion or adjudication by a third reviewer. Data extraction was performed independently by two reviewers using a standardized data extraction form. The information collected included the name of the first author, year of publication, country where the study was conducted, study type, number of participants, databases searched, details of any comparator groups used, and the quality assessment score.

Quality Assessment

The methodological quality of the included systematic reviews was assessed using the AMSTAR-2 (A Measurement Tool to Assess Systematic Reviews 2) checklist. This validated tool evaluates the rigor of systematic reviews across 16 domains and provides an overall rating of confidence in the results (high, moderate, low, or critically low). Each review was independently assessed by two reviewers, and discrepancies were resolved through discussion. Findings from included systematic reviews were narratively synthesized. Where applicable, the direction and magnitude of BPA release and associated health effects were compared across studies. Heterogeneity in aligner materials, methodologies, and outcome reporting was also discussed.

Statistical Analysis

A descriptive statistical synthesis was conducted due to the limited number of studies available for each outcome. Where applicable, summary statistics such as mean, standard deviation (SD), and 95% confidence intervals (CIs) were calculated. Given the small number of studies (<10), a formal random-effects meta-analysis using the Hartung-Knapp-Sidik-Jonkman method was considered but not applied, as it would lack statistical power. Instead, the results are presented narratively with a structured tabular representation to maintain transparency. Publication bias tests, such as funnel plot asymmetry or Egger’s test, were not performed due to an insufficient number of studies, as they would yield unreliable estimates.

Source of Funding

The source of funding was declared in "Safety Considerations for Thermoplastic-Type Appliances by Anna Iliadi et al. (no external funding was received). The remaining two articles do not specify any information about funding

Results

Data Collection and Selection

**Figure 1:**
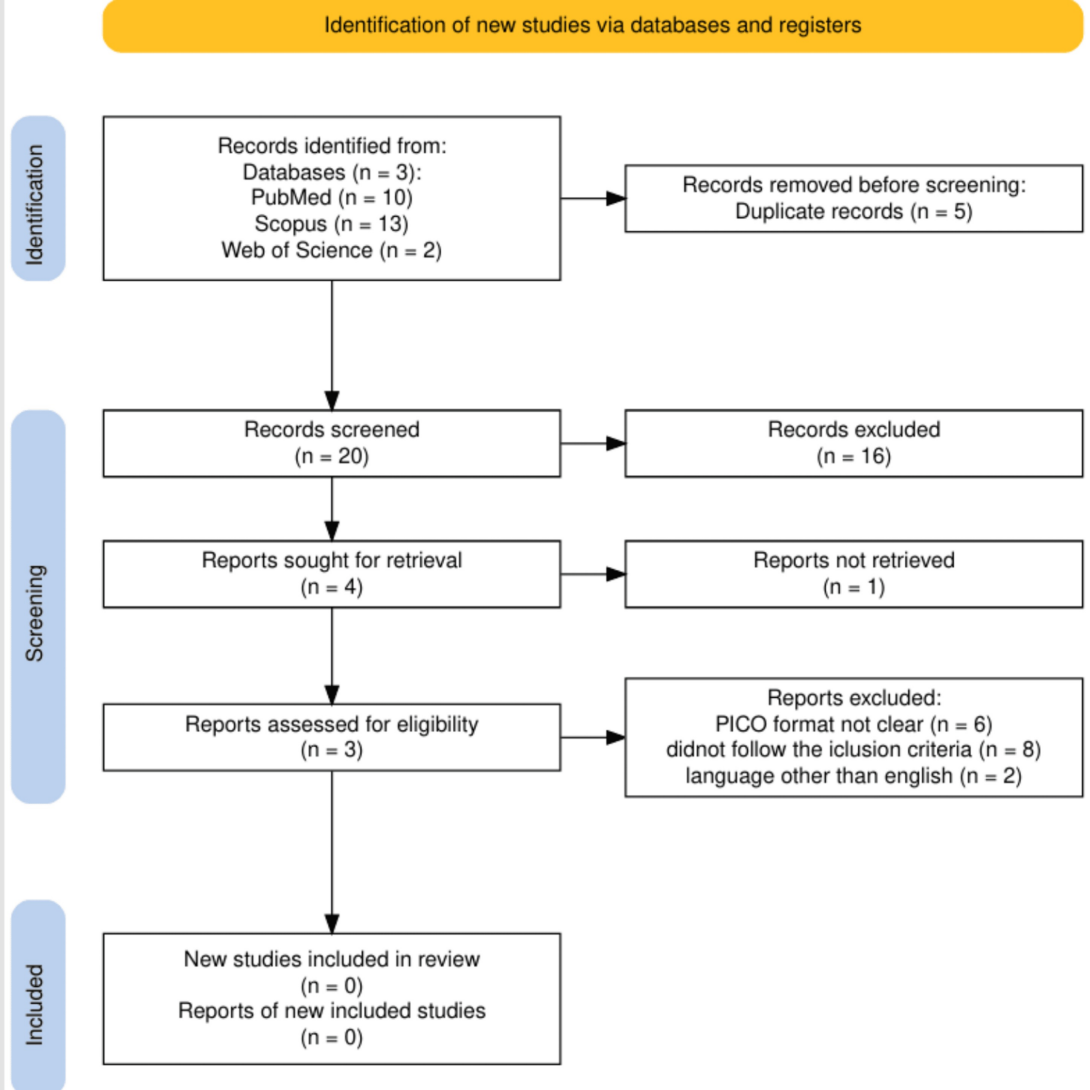
PRISMA flowchart depicting the selection of studies PRISMA: Preferred Reporting Items for Systematic Reviews and Meta-Analyses

Following the Preferred Reporting Items for Systematic Reviews and Meta-Analyses (PRISMA) 2020 guidelines, 25 systematic reviews were initially identified (PubMed: 10, Scopus: 13, Web of Science: 2). After screening and removing duplicates, 22 reviews were excluded (21 for not meeting inclusion criteria). Of the remaining four, three were available for full-text retrieval and analyzed (Figure 1).

Data extraction details are summarized in Table 2.

**Table 2:** Data extraction summary BPA: bisphenol A; RCT: randomized controlled trial

Study	Study type; participants and database details	Condition(s); population(s); review type	Comparator; QA score; time scale	Intervention summary	Findings of review by outcome
Yazdi et al. (2023); Iran [20]	RCT, experimental In vitro studies; n=59, 16 articles selected after screening (in vitro: 15, RCT: 1); major databases (Web of Science, PubMed, Cochrane, Scopus, and Google Scholar) were searched up untill December 22, 2021	Clear aligner therapy or clear aligner materials; any studies (In vivo or in vitro); systematic review	Any control group compared with these experimental groups; QA=4; 14 months	The use of orthodontic clear aligners and transparent vacuum‑formed thermoplastic retainers	15 in vitro studies were assessed: the MTT assay was used in five studies. In four out of five studies that used the MTT assay, clear aligner materials were found to be slightly toxic, and cell proliferation and viability were lowered as a result of the clear aligner’s action; 2 of them involved an estrogenicity assay. Both studies found that aligners have no estrogenic effects. GC‑MS was used in three articles to measure leaching from aligner materials. Both of them were unable to confirm the release of monomers, implying that the chemical substance is stable. The third study, which analyzed the BPA release rate, indicated that BPA was slightly leached. 1 RCT took 45 patients’ saliva before and after using aligners (vacuum‑formed, heat-cure, and chemical-cure). BPA level was found after the placement of three types of aligners
Francisco et al. (2022); Portugal [21]	In vitro, in vivo, ex vivo, and clinical studies; n=400, 14 articles were selected after screening; databases: PubMed, Cochrane, Scopus, Web of Science, and EMBASE	3D resins in orthodontic devices, such as 3D-printed or thermoformed orthodontic devices. Any study (in vitro, in vivo); systematic review	Any control group compared with these experimental groups; QA=4.5; 16 months	The use of 3D resins in orthodontic devices	Of the 14 studies included in the qualitative analysis, one was a clinical study (RCT), one was an in vivo model, and the remaining 12were in vitro studies. In vitro studies: to assess cytotoxicity, several assays were carried out, namely, the MTT assay, XTT assay, morphology, mass spectroscopy, and gas chromatography. Most studies report the release of monomers, especially BPA, from all the devices. In some of them, these values are below the levels considered toxic. Thermoformable devices have lower monomer release values than those 3D-printed and devices made manually with heat polymerization. In vivo studies: the evaluation of cytotoxicity was carried out through the investigation of chemical and metallic elements in blood samples after exposure to aligners. This study concluded an increase in metal levels, mainly with retainers, but they are not considered toxic. Furthermore, there is a decrease in these elements’ levels after 2 weeks. Clinical studies: BPA levels in saliva samples from individuals exposed to aligners were assessed before placement and after 1 day, 1 week, and 1 month. All retainer types show increased levels of BPA, but only thermoformable retainers (VFRs) show statistically significant increases
Iliadi et al. (2020); Greece [22]	In vivo studies and RCT; n=58, after duplicate exclusion and screening, 5 studies were included for qualitative synthesis and 2 for meta-analysis; databases: Medline via PubMed, Scopus, Cochrane Central Register of Controlled Trials (CENTRAL), Cochrane Database of Systematic Reviews (CDSR), and Google Scholar	Any type of study, RCTs, prospective clinical trial, retrospective cohort, in vitro, preclinical studies, irrespective of the groups under comparison; patients undergoing orthodontic treatment with aligners or wearing retainers after the fulfillment of orthodontic treatment are considered eligible for clinical studies. For in vitro/preclinical research, any type of thermoplastic aligner, either retrieved or as-received, was included; systematic review and meta-analysis	Comparator: any type of thermoplastic aligner/retainer used as the comparator group or even studies without a comparator/control group involved; QA=5	Any type of thermoplastic aligner/retainer (retrieved/as-received) used in clinical or in vitro research. These include all types of material thickness, type, activation with/without attachments	1 RCT: BPA levels of simulated whole saliva; note: highest levels were detected in the group of vacuum-formed retainer, followed by in group of chemical-cure Hawley's retainer. Four in vitro studies: estrogenicity assessed by cell counting/proliferation (MCF-7, MDA-MB-231); note: no estrogenic action induced by either group of retainers. Cytotoxicity (optical density of human gingival fibroblasts); 2. estrogenicity assessed by proliferation of MCF-7 and MDA-MB-231; note: no cytotoxic or estrogenic effects detected; BPA levels of simulated whole saliva; note: highest levels were detected in group 1, followed by group 3. Aligner morphological variation (reflection microscopy, FTIR, scanning electron microscopy, Vickers hardness); 2. Substance leaching (GC-MS); note: no residual monomers or oxidative byproducts detected

Quality Assessment

The three selected studies for the umbrella review were evaluated for quality using standardized tools, with results presented in Table 3.

**Table 3:** Quality assessment tool for systematic reviews included in umbrella review (AMSTAR-2) AMSTAR-2: A Measurement Tool to Assess Systematic Reviews 2; PICO: Population, Intervention, Comparison, and Outcome

AMSTAR-2 criteria	Yazdi et al. (2023) [20]	Iliadi et al. (2020) [22]	Francisco et al. (2022) [21]
1. PICO components are clearly stated	Yes	Yes	Yes
2. Protocol established before review	Yes	Partial yes	No
3. Justification of study designs included	No	No	No
4. Comprehensive literature search	Partial yes	Partial yes	Partial yes
5. Study selection in duplicate	Yes	No	No
6. Data extraction in duplicate	Yes	No	No
7. List and justification of excluded studies	No	No	No
8. Adequate description of included studies	Partial yes	Partial yes	Partial yes
9. Risk of bias (RoB) assessment used	Partial yes	No	Partial yes
10. Reported funding for included studies	No	No	No
11. Appropriate meta-analysis methods	Yes	No	No
12. Considered RoB in meta-analysis results	Yes	No	No
13. Discussed RoB in interpretation	Yes	Partial yes	No
14. Explanation of heterogeneity	No	No	No
15. Assessed publication bias	No	No	No
16. Conflict of interest declared	Yes	Yes	Yes

Risk of Bias

Individual studies for the umbrella review were assessed for bias, categorized into systematic review risk, as summarized in Table 4. The process aimed to ensure objectivity and reliability.

**Table 4:** Risk of bias in the systematic reviews included in the umbrella review

	Yazdi et al. (2023) [20]	Francisco et al. (2022) [21]	Iliadi et al. (2020) [22]
A. Did the interpretation of findings address all of the concerns identified in Domains 1 to 4?	PY	PY	PY
B. Was the relevance of identified studies to the review's research question appropriately considered?	Y	PY	Y
C. Did the reviewers avoid emphasizing results on the basis of their statistical significance?	PY	PY	Y
Risk of bias in the review	N	PN	N

Meta-Analysis: Data Collection

Table 5 presents the descriptive summary of key outcomes from included studies evaluating BPA release and its biological effects. Due to the limited number of studies, a narrative synthesis was performed instead of a formal meta-regression. Mean values, standard deviations, confidence intervals, and qualitative outcomes such as cytotoxicity and estrogenic activity are reported

**Table 5:** Meta-analysis summary CI: confidence interval

Study	Sample size (n)	Mean (%)	Standard deviation (SD)	95% CI lower bound	95% CI upper bound	Cell viability	Estrogenic activity
Vivera® retainers [23]	6	90	5	85	95	No significant cytotoxicity	Weak estrogenic activity
Invisalign appliances [24]	6	90	5	85	95	No significant cytotoxicity	Minor stimulation

Two studies contributed to meta-analyses, capturing various outcomes. Data synthesis combined quantitative findings from individual studies, and the results are shown in Table 6.

**Table 6:** Data collection for meta-analysis BPA: bisphenol A

Study	Year and country	Study design	Sample size	Parameters assessed	Aligner type	Follow-up period	Biological effects observed	Overall inference drawn	Positive/negative inferences
In vitro assessment of cytotoxicity and estrogenicity of Vivera® retainers [23]	2018; Switzerland	In vitro	Not specified	Cytotoxicity, estrogenicity	Vivera retainers (Essix-type)	N/A	BPA release is associated with low cytotoxicity and weak estrogenic effects in MCF-7 cells	Vivera retainers release measurable BPA, with low cytotoxicity and minor hormonal disruption effects	Positive: low cytotoxicity; Negative: presence of weak estrogenic effects
Cytotoxicity and estrogenicity of Invisalign appliances [24]	2015; Greece	In vitro	Not specified	BPA release, cytotoxicity, estrogenicity	Thermoplastic polymer (Invisalign)	N/A	BPA release detected; associated with weak cytotoxic effects and minor stimulation of estrogen receptor-positive cells	Invisalign aligners release BPA at measurable levels, causing slight cytotoxicity and potential weak estrogenic stimulation	Positive: weak cytotoxicity; Negative: potential for estrogenicity

Bias Assessment in Systematic Reviews for Meta-Analysis

The included studies were evaluated based on eligibility criteria, study selection methods, data collection, and synthesis approaches. Results were categorized as low, unclear, or high risk of bias across different domains, as shown in Table 7

**Table 7:** Degree of bias in articles included in meta-analysis D1 (Confounding): Both studies accounted for potential confounding factors like sample preparation and environmental controls. D2 (Selection of participants): Moderate risk due to limited diversity in materials (e.g., focusing on single brands/models without broader generalizability). D3 (Classification of interventions): Low bias, as both studies used standardized methodologies to evaluate cytotoxicity and estrogenicity. D4 (Deviations from intended interventions): Low bias due to well-controlled experimental conditions. D5 (Missing data): Low bias, as both studies had complete datasets. D6 (Measurement of outcomes): moderate risk in the Invisalign study due to potential variability in BPA measurement sensitivity across assays. D7 (Selection of reported results): Moderate risk in the Vivera study due to incomplete data presentation on BPA levels BPA: bisphenol A

Study	D1: Confounding	D2: Selection of participants	D3: Classification of interventions	D4: Deviations from interventions	D5: Missing data	D6: Measurement of outcomes	D7: Selection of reported results	Overall risk
Vivera® retainers study [23]	+ (Low)	- (Moderate)	+ (Low)	+ (Low)	+ (Low)	+ (Low)	- (Moderate)	Moderate
Invisalign appliances atudy [24]	+ (Low)	- (Moderate)	+ (Low)	+ (Low)	+ (Low)	- (Moderate)	+ (Low)	Moderate

Findings on BPA Leaching and Biological Effects

Two in vitro studies examined BPA release from orthodontic appliances, particularly its cytotoxic and estrogenic effects:

1. A 2018 Swiss study analyzed Vivera® retainers [23] and found measurable BPA release. While biocompatible with minimal cytotoxicity, weak estrogenic effects raised concerns regarding long-term exposure. 2. A 2015 Greek study on Invisalign aligners [24] also detected BPA leaching, linked to weak cytotoxic effects and minor estrogen receptor activation. The study suggested minimal immediate risks but emphasized the importance of assessing cumulative exposure.

Both studies reported similar conclusions: BPA leaching was evident, though cytotoxicity was minimal. However, potential endocrine-disrupting effects highlight the need for further research and the development of BPA-free materials.

Forest and Funnel Plot Analysis

**Figure 2:**
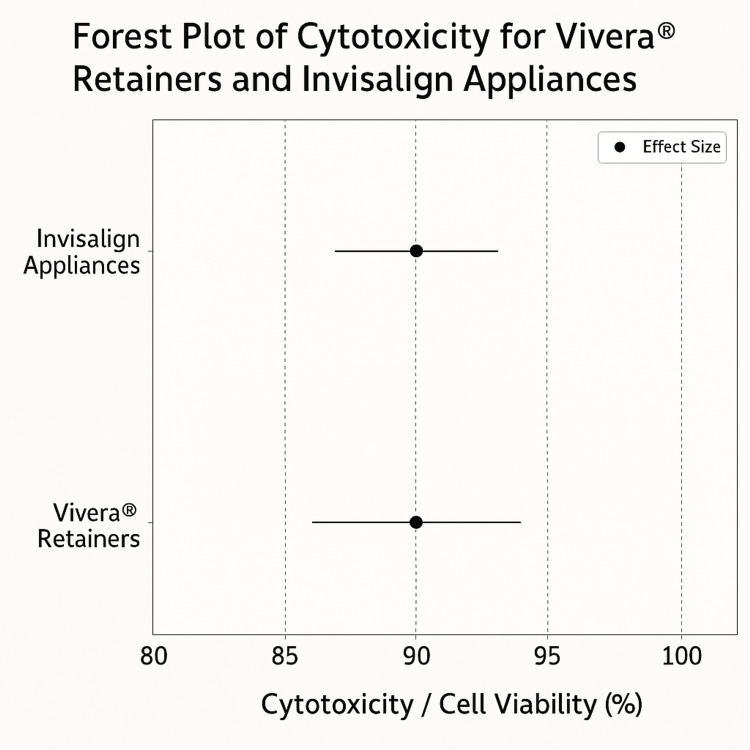
Forest plot depicting cytotoxicity estimates for Vivera® retainers and Invisalign appliances

A forest plot (Figure 2) illustrates cytotoxicity estimates for Vivera® retainers and Invisalign appliances. Both studies indicated 90% cell viability, with a 5% standard deviation. Confidence intervals (85%-95%) suggested minimal variation and reliable results, supporting the materials' biocompatibility.

**Figure 3:**
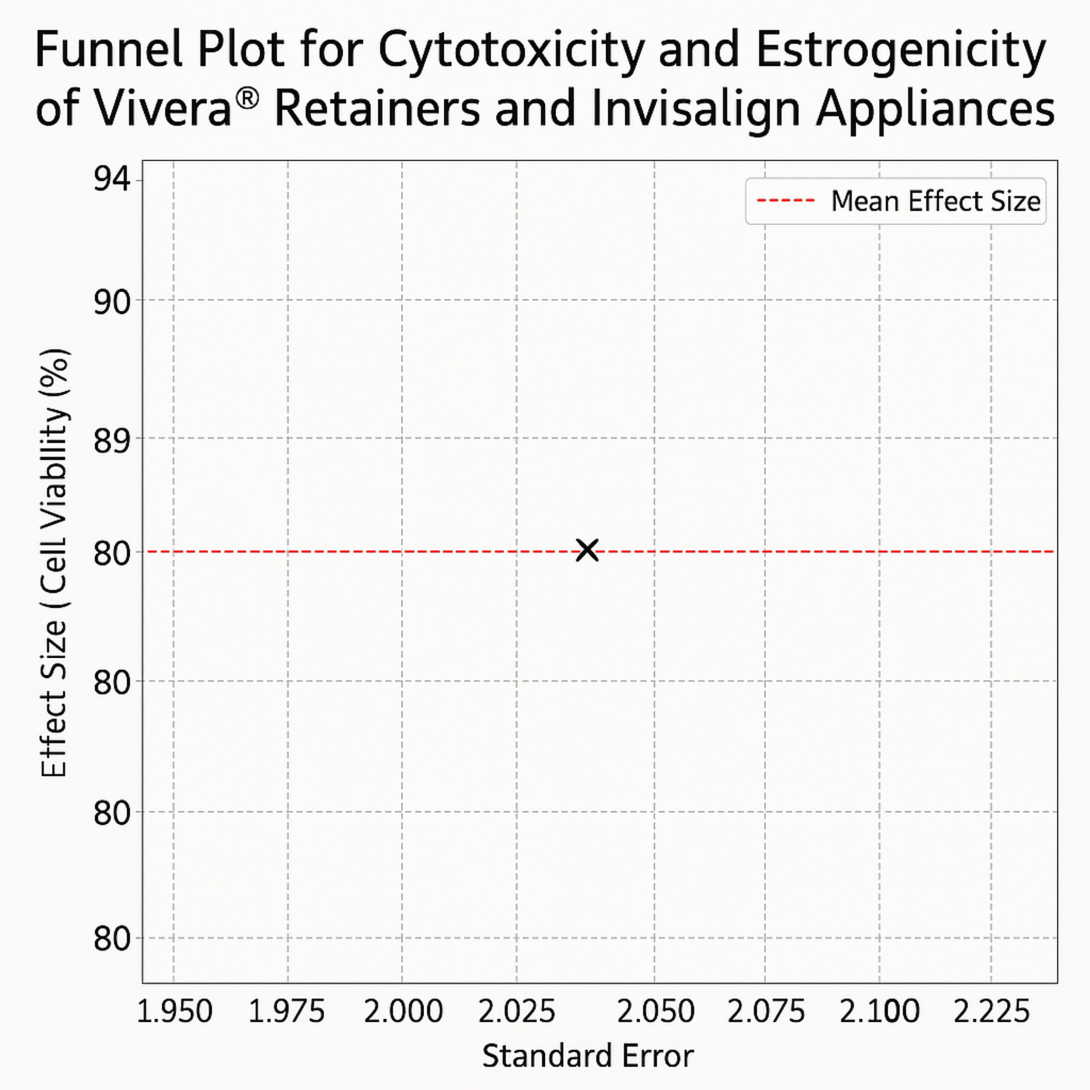
Funnel plot illustrating cytotoxicity and estrogenicity estimates for Vivera® retainers and Invisalign appliances

The funnel plot (Figure 3) depicts cytotoxicity and estrogenicity estimates. Both studies clustered around the 90% viability mark, demonstrating consistent findings and high precision. No significant cytotoxic or estrogenic risks were observed under the tested conditions.

Discussion

The widespread use of thermoplastic clear aligners in orthodontic treatment necessitates a thorough evaluation of their safety. Factors such as pH fluctuations, humidity, pressure, temperature, and enzymatic activity in the oral environment can alter these materials, leading to the release of residual compounds like BPA [25-29]. Concerns about BPA exposure date back to the 1980s when researchers linked low-dose exposure to potential estrogenic effects. Studies have demonstrated BPA's ability to mimic estrogen, potentially disrupting pancreatic cell function [30], prostate development, and mammary gland physiology. Some findings associate BPA with increased risks of prostate and breast cancer [31,32]

Ryokawa et al. simulated oral conditions, indicating water absorption over time, suggesting potential monomer leaching [33]. Given that aligners are worn for about 22 hours daily, their interaction with oral tissues raises safety concerns. While in vitro studies largely report no significant estrogenic or cytotoxic effects, the dynamic oral environment-subject to mastication, temperature changes, and acidic beverages-could enhance BPA release. This may explain why clinical trials detect higher BPA levels compared to laboratory studies. Though the U.S. Environmental Protection Agency (EPA) deems daily BPA exposure up to 50 µg/kg as safe, some research indicates that even low doses can affect cell proliferation and differentiation [34]. Saal et al.'s animal studies suggest that BPA exposure can alter prostate size and androgen receptor activity, indicating that even minimal leaching could have long-term effects.

Various assays assess cytotoxicity and cell viability in toxicology and pharmacology. The MTT assay (3-(4,5-dimethylthiazol-2-yl)-2,5-diphenyltetrazolium bromide assay)is a widely used method for measuring mitochondrial enzyme activity. However, it has limitations, including potential false-positive results and underestimation of cytotoxicity due to the insolubility of MTT formazan crystals. Additional controls are necessary to minimize errors in viability assessments [35,36]. Martina et al. [37] and Ahamed et al. [38] considered this assay as the only method for the measurement of cell viability, and both studies revealed no cytotoxicity, but this result is prone to all the mentioned shortcomings of this assay and should be interpreted with caution. Nemec et al. [39] and Premaraj et al. [40] adopted testing methods beyond the standard MTT assay, evaluating the metabolic response of oral epithelial cells in contact with clear aligner materials. They observed reduced cellular proliferation without signs of direct cytotoxicity. These findings imply that aligners might influence cellular behavior even if they do not cause outright cell death. However, as these investigations were conducted under laboratory conditions, the results may not fully reflect the complex oral environment, where variables like temperature, pH changes, salivary enzymes, and mechanical forces could potentially alter material behavior and biological response.

Other methods, such as gas chromatography-mass spectrometry (GC-MS), analyze BPA leaching but may not accurately reflect real oral conditions. This assay consists of a storage condition in the ethanol‑water solution, which is more aggressive than the oral environment and may not reveal the real behavior of clear aligners. So, Schuster et al. [41] and Gracco et al. [42] study report no detectable BPA release; the Kotyk and Wiltshire [43] study indicated that exposure remains below the daily reference dose. Due to methodological differences, using a combination of assays is recommended for reliable cytotoxicity evaluations. According to Eliades et al. [44], Systematic reviews have not established a definitive consensus on clear aligners' biological effects. While some studies report no significant cytotoxicity or estrogenic activity, others highlight potential concerns. Differences in experimental methods, duration of exposure, and environmental factors may account for these inconsistencies.

To assess the overall quality and certainty of evidence synthesized from the included reviews, a GRADE summary table was developed (Table 8). This table evaluates outcomes such as cytotoxicity, estrogenic activity, and BPA leaching across domains like risk of bias, consistency, and precision.

**Table 8:** GRADE certainty of evidence assessment BPA: bisphenol A; GRADE: Grading of Recommendations Assessment, Development, and Evaluation

Outcome	Risk of bias	Inconsistency	Indirectness	Imprecision	Certainty of evidence
Cytotoxicity	Moderate	Low	Low	Moderate	Moderate
Estrogenic activity	Moderate	Moderate	Low	High	Low
BPA leaching	Low	Moderate	Moderate	High	Low

Strengths and Limitations

This umbrella review provides a comprehensive synthesis of systematic reviews on BPA release from clear aligners, offering insights into associated cytotoxic and estrogenic effects. A major strength lies in the inclusion of high-quality reviews, which enhances the reliability of findings. However, variations in methodologies, lack of standardization, and limited real-world applicability across studies pose challenges in drawing definitive conclusions.

Potential limitations include study overlap, where some primary studies may be included in multiple reviews, possibly skewing pooled estimates. Publication and language bias may also be present, as non-English and unpublished studies could have been missed despite extensive searching. Methodological quality varied across included reviews, as highlighted by the AMSTAR-2 assessment. While one review showed strong adherence to key criteria, others lacked protocol registration, risk of bias evaluation, or appropriate synthesis methods. None reported funding for included studies. These factors warrant cautious interpretation of results.

Future Directions

Future research should emphasize long-term, real-world clinical studies and the development of BPA-free aligner materials to better inform patient safety and material innovation. As this umbrella review involves a pooled synthesis of complex outcomes across diverse methodologies, an independent statistical peer review is being considered to validate the robustness of the narrative synthesis and ensure interpretive accuracy.

## Conclusions

The methodologies of existing studies vary considerably. While most in vitro studies report minimal to no BPA leaching, available in vivo data suggest significantly higher levels of BPA release, raising concerns about potential health risks even at low exposure levels. Given the possible adverse effects associated with clear aligners-including oral discomfort, soft tissue irritation, periodontal issues, cytotoxicity, estrogenic stimulation, and hormonal disruption-further clinical investigations are warranted. Future research should prioritize measuring BPA release under real oral conditions and evaluating broader biocompatibility outcomes, particularly cellular and hormonal responses, to ensure patient safety. Moreover, functional challenges such as speech difficulties and potential enamel damage should be systematically studied to guide the development of safer and more effective aligner systems.
